# Efficacy and safety of bisphosphonates in pediatric glucocorticoid-induced osteoporosis: a meta-analysis and pharmacovigilance study

**DOI:** 10.3389/fped.2025.1571381

**Published:** 2025-07-07

**Authors:** Xiaolin Xu, Luquan Wang, Mengyang Yang, Yan Li, Changqing Yang, Dong Mei, Peng Guo, Huawei Mao

**Affiliations:** ^1^ Department of Immunity, Beijing Children’s Hospital, National Center for Children’s Health, Capital Medical University, Beijing, China; ^2^ Department of Pharmacy, Beijing Children’s Hospital, National Center for Children’s Health, Capital Medical University, Beijing, China; ^3^ School of Basic Medicine and Clinical Pharmacy, China Pharmaceutical University, Nanjing, China; ^4^ Ministry of Education Key Laboratory of Major Diseases in Children; ^5^ Department of Nephrology and Rheumatology, Children’s Hospital of Xinjiang Uygur Autonomous Region, Xinjiang Hospital of Beijing Children’s Hospital, Xinjiang, China

**Keywords:** bisphosphonates, osteoporosis, glucocorticoid, adverse reactions, meta-analysis, pediatrics

## Abstract

**Objectives:**

The prevalence of glucocorticoids (GCs) administration in pediatric populations has resulted in numerous adverse reactions, notably osteoporosis. Given its role in managing glucocorticoid-induced osteoporosis, the efficacy and safety of bisphosphonates hold considerable importance. This study conducted a meta-analysis by systematically reviewing and incorporating relevant literature on the efficacy and safety of bisphosphonates in the management of osteoporosis or bone infarction induced by GCs therapy in pediatric populations. Additionally, the analysis of potential adverse reactions was augmented by utilizing real-world data from the FAERS database. The primary objective of this study is to offer insights and guidance for the treatment of glucocorticoid induced osteoporosis in pediatric patients.

**Methods:**

A meta-analysis was performed on existing literature to assess the efficacy and safety of bisphosphonates for managing glucocorticoid-induced osteoporosis. Additionally, a retrospective pharmacovigilances study was carried out to investigate adverse reactions and medication variations in pediatric patients with glucocorticoid-induced osteoporosis, using data from the FDA Adverse Event Reporting System (FAERS) database between Q1 2004 and Q4 2023.

**Results:**

The meta-analysis incorporated a total of 14 articles encompassing 572 patients. The findings of this study indicate that bisphosphonate therapy is more effective in enhancing bone mineral density (BMD) and BMD *Z*-scores in children compared to the control group, albeit with a heightened risk of adverse reactions. Furthermore, there was no significant disparity observed between the impact of bisphosphonate treatment and control groups on fracture outcomes. Subsequently, in the ensuing Pharmacovigilance investigation, 668 instances of adverse reactions associated with bisphosphonates are analyzed. The findings indicated that the most prevalent adverse reactions, as evidenced by the highest number of positive signals were various examinations, musculoskeletal and connective tissue diseases, injuries, poisoning and operational complications, as well as systemic diseases and reactions at the administration site.

**Conclusions:**

This study conducted a comprehensive analysis of the efficacy and safety of bisphosphonates in the treatment of osteoporosis caused by GCs use in pediatric patients, laying the groundwork for future research. Nevertheless, the constraints of retrospective studies highlight the need for additional investigation through prospective studies.

## 1 Introduction

Osteoporosis is a condition characterized by heightened bone fragility and susceptibility to fractures. The study of secondary osteoporosis in pediatric populations is gaining prominence alongside primary hereditary osteoporosis. The primary objectives of treatment include the prevention of fractures, enhancement of bone mass, augmentation of trabecular and cortical thickness, restoration of vertebral fractures, correction of skeletal deformities, and enhancement of mobility, independence, and quality of life (1).

Glucocorticoids (GCs) are frequently recognized as a primary contributor to secondary osteoporosis. The resultant skeletal disorder characterized by decreased bone density and an elevated risk of fractures due to prolonged glucocorticoid therapy is referred to as glucocorticoid-induced osteoporosis. Although physiological doses of glucocorticoids are essential for osteoblast differentiation, high doses of glucocorticoids can promote osteoclast activity and suppress the function of both osteoblasts and osteoclasts. This results in reduced bone density and compromised bone microarchitecture. In pediatric and adolescent populations, these medications are primarily prescribed for the management of conditions such as asthma, rheumatologic diseases, and autoimmune disorders. Adolescents, being in the critical phase of peak bone mass acquisition, are consequently more vulnerable to the detrimental skeletal effects associated with glucocorticoid therapy. This is especially true for those receiving long-term glucocorticoid therapy or systemic glucocorticoid treatment during puberty, as they are at an elevated risk of developing glucocorticoid-induced osteoporosis (2–6). Presently, the primary therapeutic modalities encompass both non-pharmacologic and pharmacologic interventions. Non-pharmacological strategies consist of nutritional supplementation, adherence to a balanced diet, and exercise management. Pharmacological treatments predominantly consist of bisphosphonates, selective estrogen receptor modulators, calcitonin, and molecularly targeted drugs (7), which primarily function by suppressing osteoclast activity and decreasing bone turnover. Moreover, osteonecrosis is a recognized complication associated with glucocorticoid therapy. This condition is characterized by the necrosis of bone component cells resulting from an interruption in blood supply. Pharmacological interventions, particularly the administration of bisphosphonates,constitute a fundamental therapeutic approach for managing this condition. In clinical practice, numerous pediatric patients have encountered the adverse reaction of bone infarction subsequent to glucocorticoid administration. Given that bone infarction is a form of osteonecrosis, the effectiveness and safety of pharmacological interventions for this condition have become a focal point of our investigation.

Bisphosphonates are the most commonly utilized among the aforementioned drugs (8, 9). Their mechanism of action involves binding to bone minerals, uptake by osteoclasts during bone resorption, and subsequent inhibition of osteoclast activity (10). Bisphosphonates, a category of pharmaceutical agents, function by impeding bone resorption through various pathways. Extensive research has demonstrated the safety and efficacy of bisphosphonates, as evidenced by their ability to increase bone mineral density and decrease fracture risk, ultimately leading to a reduction in mortality rates and enhancement of overall quality of life (11).

Bisphosphonates possess the capacity to impede osteoclast activity through the inhibition of farnesyl pyrophosphate synthetase within osteoclasts, thereby disrupting the geranylation of geranyl and resulting in osteoclast inactivation. This mechanism is accountable for suppressing the nitrogen-containing bisphosphonates (N-BP) of bone resorption by osteoclasts and decreasing bone turnover in order to mitigate the risk of fractures (12, 13). A systematic review comprising 48 studies involving adult patients found that long-term administration of alendronate and zoledronate could effectively decrease the fracture risk in women diagnosed with osteoporosis (14). Nevertheless, the potential adverse effects of bisphosphonates, including gastrointestinal issues, kidney damage, bone necrosis, and eye inflammation, have garnered significant attention in the medical community (15–17). Currently, there is no approved indication for bisphosphonate use, yet it continues to be utilized off-label. The decision to prescribe this medication necessitates a thorough evaluation of the trade-off between its therapeutic benefits and potential adverse effects.

This study utilized a meta-analysis of prior research to investigate the effectiveness and safety of bisphosphonates in treating osteoporosis or bone infarction resulting from hormone use in pediatric patients. Additionally, real-world pharmacovigilance data from the Food and Drug Administration's adverse event reporting system (FAERS) was employed to examine reported incidents of adverse reactions associated with bisphosphonate use in clinical settings.

## 2 Methods

### 2.1 Research design

Initially, a meta-analysis was performed on extant literature to examine the effectiveness and safety of bisphosphonates for managing glucocorticoid-induced osteoporosis and Bone Infarction. Furthermore, retrospective data mining analysis utilizing the FDA FAERS database was conducted to address limitations in current studies regarding adverse reactions and variations among different medications.

### 2.2 Systematic evaluation procedures

#### 2.2.1 Access to literature

**Figure 1:**
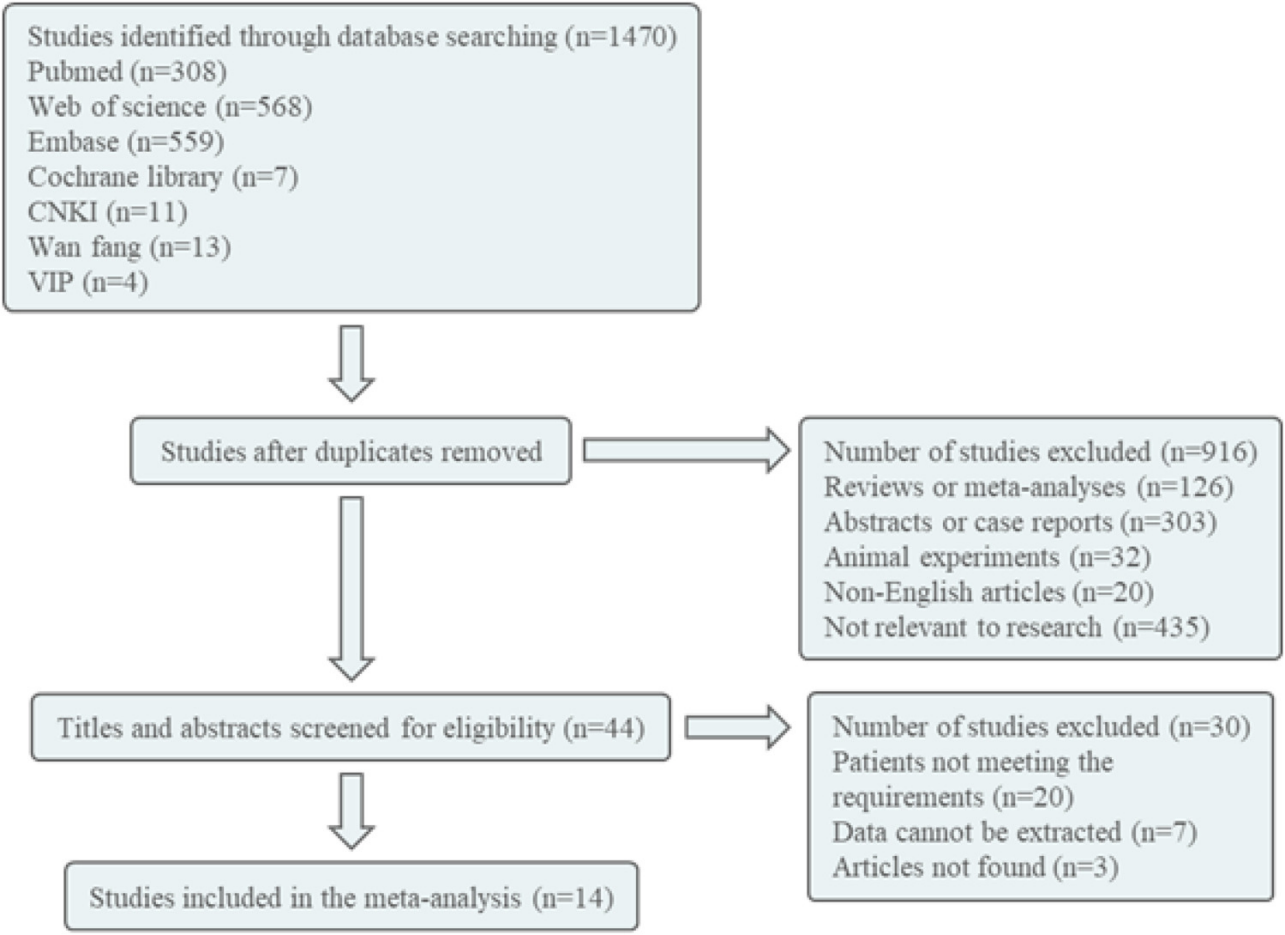
Document screening flow chart.

The retrieval database utilized in this study includes Pubmed, Embase, Cochrane Library, Web of Science, HowNet, VIP, and Wanfang. The English search terms employed are Diphosphates Bisphosphonates, Bisphosphonate, Alendronate, Etidronate, Zoledronate, Clodronate, Pamidronate, Tiludronate, Neridronate, Olpadronate, Risedronate, Ibandronate, Child, Children, Osteoporosis, bone loss, and bone infarct. The Chinese search term used is bisphosphonate, children, osteoporosis/bone loss/bone infarction, with the search conducted on October 7, 2023. The literature is initially screened by reviewing the title and abstract. Following this preliminary screening process, any literature deemed unsuitable will be excluded after a thorough examination of the full text, with the remaining literature being included in the study (Figure 1).

#### 2.2.2 Criteria for inclusion and exclusion of literature

(1) Inclusion criteria.
  A. Subjects: Patients with a documented history of glucocorticoid use within the past year, who subsequently received a diagnosis of osteoporosis or bone infarction following such use, were included in the study.
  B. Intervention mode: bisphosphonate, including Alendronate Etidronate, Zoledronate, Clodronate, Pamidronate, Tiludronate, Neridronate, Olpadronate, Risedronate, Ibandronate, etc. The control group is the placebo group or the drug itself before and after control.
  C. Outcome: (a) Bone mineral density (BMD) and BMD *z*-scores; (b) Frame Outcomes; (c) Paint and mobility outcomes; (d) Quality of Life; (e) Muscle strength, etc.
  D. Research type: RCT or cohort study.
(2) Exclusion criteria.
  A. The patients exhibited primary osteoporosis and Primary Bone Infarction, encompassing conditions such as osteogenesis imperfecta, adolescent idiopathic osteoporosis, Spontaneous Bone Infarction, Posttraumatic Bone Infarction, and other hereditary diseases.
  B. Secondary osteoporosis caused by non-GCs treatment, such as too little exercise, muscle weakness, chemotherapy drugs and other bone destruction, Secondary osteoporosis.
  C. Research on incomplete data or inability to extract data;
  D. Meta, review, Meeting summary, animal test, case report;
  E. Non-Chinese/English literature.

#### 2.2.3 Quality assessment and data extraction

The modified Jadad scoring scale was utilized to evaluate the quality of randomized controlled trials, based on four criteria: random sequence generation, allocation concealment, blinding procedures, and participant attrition. The total score of the randomized controlled trials was 7 points, with scores ranging from 1 to 3 indicating low quality and scores from 4 to 7 indicating high quality. The Newcastle-Ottawa Scale (NOS) was employed to assess the quality of cohort and case-control studies across three dimensions: selection of the study population, comparability between groups, and measurement of the outcomes, with a total possible score of 9 points, categorized as poor (0–3), fair (4–6), or good (7–9). For cross-sectional studies, the Joanna Briggs Institute (JBI) scale, published by the Australian Centre for Evidence-Based Nursing, was used. This scale comprises 10 items, with a total possible score of 20 points.

Literature screening and data extraction: Two researchers conducted a comprehensive review of the literature, independently gathering data, evaluating quality, and verifying results. Any discrepancies were addressed through discussion and resolution, or by involving a third researcher for input. Data extraction encompassed various elements, such as basic information (e.g., authors, publication date, case count), interventions (e.g., program details, treatment regimen), and outcome indicators.

#### 2.2.4 Statistical processing

The data analysis was conducted using Stata15.1 software, utilizing the Weighted Mean Difference (WMD) and Hazard Ratio (RR) as statistical measures, with effect sizes expressed through 95% confidence intervals (CI). Heterogeneity testing was performed on each outcome, with the random effect model utilized if the I^2^ statistic was greater than or equal to 50%, and the fixed effect model used otherwise. Sensitivity analysis was conducted for all outcomes, and publication bias was assessed through the Begg's test. The observed discrepancy was found to be statistically significant at a significance level of *P* < 0.05.

### 2.3 Pharmacovigilance study procedures

Search the real world pharmacovigilance data related to bisphosphonates in FAERS from January 1, 2004 to December 31, 2023. The common names of bisphosphonates (Alendronate, Etidronate, Zoledronate, Clodronate, Pamidronate, Risedronate, Ibandronate) are used to identify cases reported as suspected bisphosphonate. Furthermore, the study collected clinical characteristics data such as the reporter's profession (health care professional or non-health care professional), gender, age, reporting country, reporting year, and additional data as outlined in Table 1. Reports in the FAERS database are categorized using preferred terms (PTs) from the MedDRA hierarchy, including high-level terms (HLT), high-level group terms (HLGT), and system organ class (SOC) levels. For the purposes of this study, the SOC level was chosen to classify adverse events related to bisphosphonates.

**Table 1:** Characteristics of cases from FAERs database.

Category		Total		Alendronate		Etidronate		Zoledronate		Clodronate		Pamidronate		Risedronate		Ibandronate	
Data resource	FAERS	668		88		3		350		1		185		21		20	
Reporter	Health-care professional	579	86.68%	67	76.14%	3	100%	320	91.43%	1	100%	160	86.49%	14	66.67%	14	70%
	Non-health-care professional	48	7.19%	18	20.45%			17	4.86%			2	1.08%	5	23.81%	6	30%
	Not specified	41	6.14%	3	3.41%			13	3.71%			23	12.43%	2	9.52%		
Sex	Female	282	42.22%	46	52.27%			138	39.43%	1	100%	85	45.95%	9	42.86%	13	65%
	Male	363	54.34%	38	43.18%	3	100%	211	60.29%			92	49.73%	12	57.14%	7	35%
	Not specified	13	1.95%	4	4.55%			1	0.29%			8	4.32%				
Age	Mean (SD)	7.52		6.96		4.39		9.92		7		7.94		9.79		7.36	
	Min, Max	0, 17		0, 17		0.17, 7		0, 17		7, 7		0, 17		0,16		0.01, 17	
Report countries	North America	235	35.18%	35	39.77%	3	100%	134	38.29%			59	31.89%	1	4.76%	3	15%
	Europe	269	40.27%	28	31.82%			162	46.29%			60	32.43%	11	52.38%	8	40%
	Asia	51	7.63%	17	19.32%			14	4.00%			18	9.73%			2	10%
	Africa	6	0.9%	1	1.14%			5	1.43%								
	South America	4	0.60%					3	0.86%							1	5%
	Oceania	18	2.69%									18	9.73%				
	Not specified	77	11.53%	7	7.95%			24	6.86%	1	100%	30	16.22%	9	42.86%	6	30%
Reporting year	Before 2011	126	18.86%	21	23.86%			21	6.00%	1	100%	76	41.08%	5	23.81%	2	10%
	2011	24	3.59%	4	4.55%			7	2.00%			11	5.95%	1	4.76%	1	5%
	2012	38	5.69%	3	3.41%	1	33.33%	10	2.86%			23	12.43%			1	5%
	2013	16	2.40%	4	4.55%	1	33.33%	8	2.29%			1	0.54%	2	9.52%		
	2014	23	3.44%	6	6.82%			8	2.29%			6	3.24%	3	14.29%		
	2015	24	3.59%	3	3.41%			15	4.29%			6	3.24%				
	2016	41	6.14%	10	11.36%	1	33.33%	23	6.57%			7	3.78%	1	4.76%		
	2017	49	7.34%	4	4.55%			13	3.71%			21	11.35%	6	28.57%	5	25%
	2018	56	8.38%	3	3.41%			41	11.71%			10	5.41%	1	4.76%	1	5%
	2019	76	11.38%	9	10.23%			61	17.43%			4	2.16%	1	4.76%	1	5%
	2020	39	5.84%	9	10.23%			21	6.00%			5	2.70%	1	4.76%	3	15%
	2021	67	10.03%	2	2.27%			62	17.71%			2	1.08%			1	5%
	2022	39	5.84%	5	5.68%			24	6.86%			7	3.78%			3	15%
	2023	49	7.34%	5	5.68%			36	10.29%			6	3.24%			2	10%
Time to onset	0-30 days	167	25%	13	14.77%			108	30.86%			37	20.00%	6	28.57%	3	15%
	31–60 days	4	0.6%					2	0.57%			2	1.08%				
	61–90 days	3	0.45%	1	1.14%											2	10%
	91–120 days	3	0.45%	2	2.27%			1	0.29%								
	121–150 days	3	0.45%					3	0.86%								
	151–180 days	2	0.3%					1	0.29%					1	4.76%		
	181–360 days	6	0.9%	1	1.14%			4	1.14%	1	100%						
	360 days<	16	2.4%	6	6.82%			7	2%			2	1.08%	1	4.76%		
	Other	464	69.46%	65	73.86%	3	100%	224	64.00%			144	77.84%	13	61.90%	15	75%
Outcome	Life-threatening	44	6.59%	5	5.68%			25	7.14%			12	6.49%			2	10%
	Hospitalization- Initial or Prolonged	259	38.77%	29	32.95%			171	48.86%	1	100%	50	27.03%	5	23.81%	3	15%
	Disability	27	4.04%	5	5.68%	1	33.33%	14	4%			4	2.16%			3	15%
	Death	25	3.74%					11	3.14%	14	7.57%						
	Congenital anomaly	14	2.1%	11	12.5%					3	1.62%						
	Required intervention to prevent permanent	9	1.35%	4	4.55%			3	0.86%			2	9.52%				
	Impairment/Damage																
	Other	433	64.82%	55	62.50%	2	66.67%	213	60.86%	137	74.05%	16	76.19%	10	50%		

### 2.4 The role of financial resources

The funding entity did not participate in the formulation of the research design, data collection, data analysis, data interpretation, or report composition.

## 3 Results

### 3.1 Results of the systematic evaluation

#### 3.1.1 The findings of the literature search and the fundamental aspects of literature acquisition

Following the implementation of the search strategy in both Chinese and English databases, a total of 1,470 articles were identified. These articles underwent screening based on predetermined inclusion and exclusion criteria, resulting in the inclusion of 14 articles for further analysis (18–31). The document screening flow chart 1 illustrates the specific process, which included 6 randomized controlled trials (RCTs), 6 cohort studies, 1 case-control study, and 1 cross-sectional study. Among the included articles, seven were deemed of high quality and seven were of medium quality. A total of 572 patients were involved in the study, as indicated in Table 2.

**Table 2:** Characteristics of the included literature.

Author	Year	Country	Study design	Patients	Groups	Intervention	Sample size	Female (%)	Age, years	Follow- up	Outcomes	Quality
Rudge	2005	New Zealand	RCT	Long-term prednisone therapy	Bisphosphonates	Alendronate, po. 1–2 mg/kg/week	11	54.5%	8.7	12 m	BMD, BMD *Z*-scores, fracture, adverse events	6
					Control	Placebo	11	63.6%	8			
Kim	2006	Korea	RCT	Nephropathy receiving high doses of steroids	Bisphosphonates	Pamidronate, po. 125 mg/day	22	22.7%	8.5 (4.49)	3 m	BMD	5
					Control	Calcium	22	50%	8.5 (2.39)			
Rooney	2019	United Kingdom	RCT	Juvenile idiopathic arthritis, juvenile SLE, juvenile dermatomyositis, or vasculitis	Bisphosphonates	Risedronate, po. 1 mg/kg/week if ⩽30 kg) or 35 mg/week if >30 kg)	69	76.8%	12 (3.4)	12 m	BMD, BMD *Z*-scores, fracture, adverse events	7
					Control	Calcium and VD	77	71.4%	12.1 (3.5)			
Zacharin	2021	Australia	RCT	Glucocorticoid dependent Duchenne muscular dystrophy	Bisphosphonates	Zoledronate, iv. 0.025 mg/kg at 0 and 3months, 0.05 mg/kg at 6, 12 and 18months	31		10.1 (2.8)	24 m	BMD, BMD *Z*-scores, fracture, adverse events	4
					Control	Calcium and VD	31		10.1 (2.6)			
Ward	2021	Multinational	RCT	Nonmalignant conditions treated with systemic glucocorticoid	Bisphosphonates	Zoledronate, iv. 0.05 mg/kg at 0 and 6months	18	33%	13.0 (3.5)	12 m	BMD, BMD *Z*-scores, fracture, adverse events	7
					Control	Calcium and VD + placebo	16	31%	12.3 (3.4)			
Acott	2005	Canada	Case-control study	Chronic steroid therapy	Bisphosphonates	Pamidronate, 1 mg/kg/dose (maximum 90 mg), once every 2months, administered intravenously over four hours for 1 yr or 2 yr	17	47.06%	N/A	36 m	BMD *Z*-scores	5
Brown	2005	Australia	RCT	Chronic disease requiring long-term corticosteroid treatment	Bisphosphonates	Calcium carbonate 600–1200 mg daily and calcitriol 0.25 mcg daily or disodium pamidronate 1 mg/kg by intravenous infusion every 3 months	5	N/A	4–18	48 m	BMD, BMD *Z*-scores	6
					Control	calcium/vitamin D	7	N/A	4–18			
Inoue	2008	Japan	Cohort study	Children with autoimmune diseases	Bisphosphonates	5 mg intravenous alendronate delivered over a 4-h period once every 3 months	5	80%	N/A	24 m	BMD *Z*-scores, serum bone alkaline phosphatase (BAP), urinary deoxypyridinoline (DPD) levels	4
Inoue	2018	Japan	Cross-sectional study	Childhood-onset rheumatic disease who received glucocorticoid therapy	Bisphosphonates	Oral alendronate weekly (35 mg for ≥30 kg, 25 mg for 20 kg to <30 kg, 15 mg for 15 kg to <20 kg)	18	88.90%	10.9 [7.8–13.5]	36 m	BMD *Z*-scores	15
					Control	Vitamin D and calcium	21	71.40%	10.6 [8.3–11.8]			
Lim	2021	Australia	Cohort study	Glucocorticoid-induced osteoporosis	Bisphosphonates	At least 1 dose of zoledronate	27	44.44%	12.6 (2.9)	12 m	BMD, BMD *Z*-scores	5
Moretti	2022	Italy	Cohort study	Duchenne muscular dystrophy, treated with glucocorticoids	Bisphosphonates	Neridronate an intramuscular (IM) dose of 25 mg every month and daily vitamin D (600 IU) and calcium supplementation (500 mg)	8		4.75 (2.81)	12 m	BMD, BMD *Z*-scores	5
Nasomyont	2021	United States	Cohort study	Duchenne muscular dystrophy, treated with glucocorticoids	Bisphosphonates	Alendronate, 17.5 mg weekly for patients aged 5 to 7 years and 35 mg weekly for patients aged ≥8 years	52	N/A	11.8 [10.0–13.6]	12 m	Prevalence and sseverity of vertebral fractures, Back pain assessment, adverse events	5
Srinivasan	2016	United Kingdom	Cohort study	Duchenne muscular dystrophy, treated with glucocorticoids	Bisphosphonates	The regimen was 35 mg of risedronate sodium per week. Children under 20 kg in weight received a reduced dose (approximately 1 mg/kg/week)	35	0	9.2 (3.4)	4.9 y	BMD *Z*-scores, fracture	6
					Control	Vitamin D and calcium	15	0	8.8 (2.3)	6.2 y		
Tian	2020	USA	Cohort study	Duchenne muscular dystrophy, treated with glucocorticoids	Bisphosphonates	starting dose of oral alendronate for pediatric patients was 35 mg once a week (half of the adult dose of 70 mg once a week) for patients aged 8 years and older, and 17.5 mg once a week for younger patients aged 5 to 7 years	54	0	11 [9.3, 12.7]	6 y	BMD, BMD *Z*-scores	5

Note: BMD, bone mineral density; iv., intravenous; M, month; Y, year; NA, not available; po., per os, means oral; VD, vitamin D; data presented as median [25th, 75th percentile], mean (SD), or *n* (%).

#### 3.1.2 Changes in BMD

**Figure 2:**
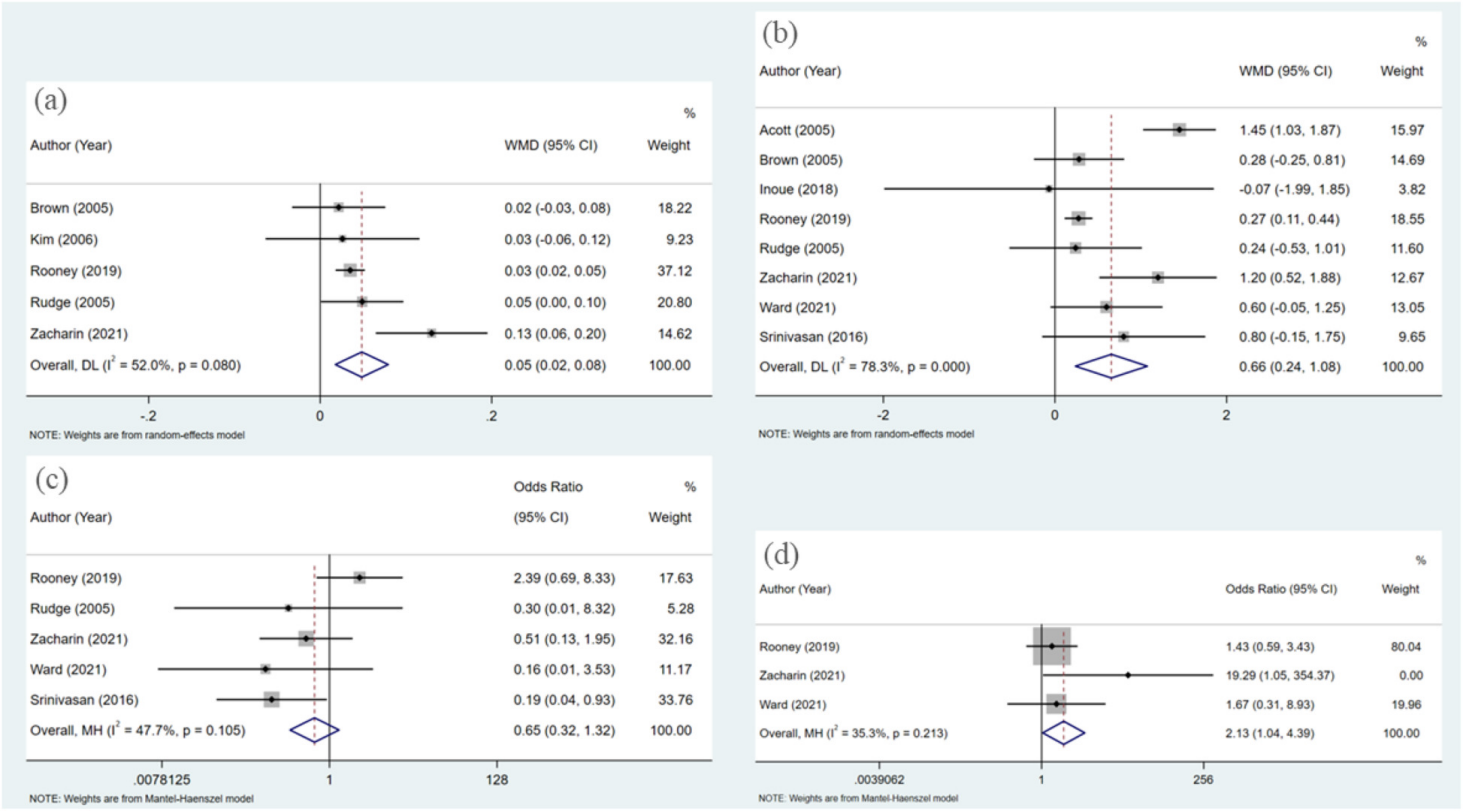
Forest plot of meta-analysis: **(a**) changes in BMD; **(b)** changes in BMD *Z*-scores; **(c)** fracture; **(d)** adverse events.

A total of five articles were incorporated into the analysis,each employing dual-energy x-ray absorptiometry (DXA) to assess of bone mineral density (BMD). The studies included a variety of bisphosphonates, specifically alendronate, pamidronate, risedronate, and zoledronate, with follow-up time spanning from 3 to 48 months. The heterogeneity test revealed statistically significant results (I^2^ = 52.0%). Therefore, a random effects model was employed for the combined analysis (Figure 2a). The weighted mean difference (WMD) was 0.049, with a 95% confidence interval (CI) of 0.018–0.079, and a *P*-value of 0.002. The results indicated statistically significant differences among the combined studies, suggesting that the improvement in bone mineral density (BMD) in pediatric patients treated with bisphosphonate was superior to that of the control group.

#### 3.1.3 Changes in BMD *Z*-scores

In the eight studies analyzed, the *Z*-score, a critical metric in the assessment of BMD, was evaluated using dual-energy x-ray absorptiometry (DXA), akin to the measurement of BMD itself. The bisphosphonates investigated within these studies included alendronate, pamidronate, risedronate, and zoledronate, with follow-up durations ranging from 6 to 48 months. The heterogeneity test yielded statistically significant results (I^2^ = 78.3%), necessitating the application of a random effects model for the aggregated analysis (Figure 2b).The weighted mean difference (WMD) was found to be 0.656, with a 95% confidence interval of (0.236, 1.076) and a *P*-value of 0.002. Significant differences were observed among the combined studies, indicating that the efficacy of bisphosphonate in improving BMD *Z*-scores of pediatric patients is superior to that of the control group.

#### 3.1.4 Fracture

The analysisincorporated a total of five articles, each of which documented the incidence of new fractures in patients during follow-up periods to evaluate the efficacy of bisphosphonate treatment in preventing fractures. The heterogeneity test revealed no statistically significant differences among these studies (I^2^ = 47.7%). Therefore, the fixed effect model was utilized for the combined analysis (Figure 2c). The odds ratio was 0.649 with a 95% confidence interval of 0.319–1.319, and a *p*-value of 0.232. These findings suggest that there was no statistically significant difference among the combined studies, indicating that the effects of bisphosphonate treatment and control groups on fracture outcomes cannot be considered to be different.

#### 3.1.5 Adverse events

Three articles were included in the analysis, and the heterogeneity test results did not show any statistically significant differences (I^2^ = 35.3%). Therefore, the fixed effect model was utilized for the combined analysis (Figure 2d). The pooled odds ratio was 2.135 with a 95% confidence interval of (1.039, 4.388) and a *p*-value of 0.039, indicating statistically significant differences among the included studies. These findings suggest that the risk of adverse reactions in pediatric patients treated with bisphosphonate is higher compared to those in the control group.

#### 3.1.6 Sensitivity analysis and publication bias

A sensitivity analysis was conducted to evaluate the potential influence of excluding individual studies on the overall findings of the study. The results indicated that none of the studies had a substantial impact on the overall results, demonstrating the stability of the findings (Table 3). Furthermore, the results of Begg's publication bias test revealed no evidence of publication bias in any of the outcomes (*P* < 0.05) (Table 4).

**Table 3:** Summary of sensitivity analysis.

Changes in BMD				Changes in BMD *Z*-scores			
Study omitted	Estimate	[95% Conf.	Interval]	Study omitted	Estimate	[95% Conf.	Interval]
Brown (2005)	0.0564	0.0171	0.0956	Acott (2005)	0.4432	0.1841	0.7023
Kim (2006)	0.052	0.0163	0.0877	Brown (2005)	0.7175	0.2213	1.2137
Rooney (2019)	0.057	0.0097	0.1042	Inoue (2018)	0.6854	0.2503	1.1205
Rudge (2005)	0.05	0.0082	0.0925	Rooney (2019)	0.7468	0.2968	1.1968
Zacharin (2021)	0.0351	0.0199	0.0503	Rudge (2005)	0.7105	0.2428	1.1782
Combined	0.0485	0.0177	0.0794	Zacharin (2021)	0.5779	0.135	1.0207
				Ward (2021)	0.663	0.1839	1.1421
				Srinivasan (2016)	0.6398	0.1844	1.0953
				Combined	0.6562	0.2364	1.0761
Fracture				Adverse events			
Study omitted	Estimate	[95% Conf.	Interval]	Study omitted	Estimate	[95% Conf.	Interval]
Rooney (2019)	0.2752	0.1018	0.7437	Rooney (2019)	4.9722	1.2278	20.1364
Rudge (2005)	0.6848	0.3338	1.4048	Zacharin (2021)	1.4752	0.6785	3.2075
Zacharin (2021)	0.7154	0.3092	1.6551	Ward (2021)	2.2516	1.0117	5.011
Ward (2021)	0.73	0.3521	1.5144	Combined	2.1349	1.0387	4.3881
Srinivasan (2016)	0.8837	0.3941	1.9815				
Combined	0.6487	0.3189	1.3194				

**Table 4:** Summary of analytical results.

Ending	Analyzing indicators	*n* of studies	WMD/OR (95% CI)	*P*	I^2^ (%)
changes in BMD	Overall	5	0.049 (0.018, 0.079)	0.002	52.0
	Sensitivity analysis		0.049 (0.018, 0.079)		
	Publication bias		Z = 0.73	0.462	
changes in BMD *Z*-scores	Overall	8	0.656 (0.236, 1.076)	0.002	78.3
	Sensitivity analysis		0.656 (0.236, 1.076)		
	Publication bias		Z = 0.37	0.711	
Fracture	Overall	5	0.649 (0.319, 1.319)	0.232	47.7
	Sensitivity analysis		0.649 (0.319, 1.319)		
	Publication bias		Z = 0.73	0.462	
Adverse events	Overall	3	2.135 (1.039, 4.388)	0.039	35.3
	Sensitivity analysis		2.135 (1.039, 4.388)		
	Publication bias		Z = 1.04	0.296	

### 3.2 Pharmacovigilance study procedures

Between January 1, 2004, and December 31, 2023, a total of 668 cases of adverse reactions associated with bisphosphonates were documented. Specifically, there were 88 cases involving alendronate, 3 cases involving etidronate, 350 cases involving zoledronate, 1 case involving clodronate, 185 cases involving pamidronate, 21 cases involving risephosphate, and 20 cases involving ibandronate. Furthermore, it is noteworthy that all patients included in the study were minors, under the age of 18. The demographic details of the patients are outlined as follows. The majority of cases were documented by healthcare professionals (86.68%). The mean age at which symptoms first appeared was 7.52 years. The prevalence of male cases slightly exceeded that of female cases (54.34% vs. 42.22%). Since 2016, there has been a rise in the number of reported cases, with Europe, Asia, and North America being the primary regions of concern. The predominant reason for hospitalization was adverse reactions, occurring in the majority of patients within one month of initiating the drug (Table 1).

In this investigation, a systematic review of the literature was performed to identify specific clinical cases associated with adverse events (AEs) reported for seven bisphosphonates through a search of the System Organ Class (SOC) database. The results showed that the four adverse reactions with the highest number of positive signals were various examinations (ROR: 20; PRR: 20; MHRA: 20; BCPNN: 18; MGPS: 58), various musculoskeletal and connective tissue diseases (ROR: 26; PRR: 26; MHRA: 26; BCPNN:23; MGPS: 86), various injuries, poisoning and operational complications (ROR: 29; PRR: 29; MHRA: 26; BCPNN: 26; MGPS: 65) and systemic diseases and various reactions at the administration site (ROR: 21; PRR: 21; MHRA: 20; BCPNN: 18; MGPS: 32) (Table 5).

**Table 5:** Statistics on the number of signals.

English name of system organ classification (SOC)	Number of signals				
	ROR	PRR	MHRA	BCPNN	MGPS
Injury, poisoning and procedural complications	29	29	26	26	65
Musculoskeletal and connective tissue disorders	26	26	26	23	87
General disorders and administration site conditions	21	21	20	18	32
Gastrointestinal disorders	8	8	8	7	22
Investigations	20	20	20	18	58
Neoplasms benign, malignant and unspecified (incl cysts and polyps)	5	5	5	4	23
Congenital, familial and genetic disorders	5	5	5	5	16
Metabolism and nutrition disorders	11	11	11	10	24
Nervous system disorders	6	6	6	6	16
Renal and urinary disorders	10	10	10	8	17
Hepatobiliary disorders	1	1	1	1	7
Respiratory, thoracic and mediastinal disorders	7	7	7	5	14
Psychiatric disorders	1	1	1	1	5
Infections and infestations	5	5	5	4	15
Ear and labyrinth disorders	2	2	2	2	5
Pregnancy, puerperium and perinatal conditions					1
Vascular disorders	4	4	4	4	14
Surgical and medical procedures					12
Skin and subcutaneous tissue disorders	1	1	1	1	5
Reproductive system and breast disorders					2
Blood and lymphatic system disorders	6	6	6	5	8
Eye disorders	1	1	1	1	3
Product issues					
Endocrine disorders					3
Cardiac disorders	3	3	3	3	7
Immune system disorders					1
Social circumstances					3

**Figure 3:**
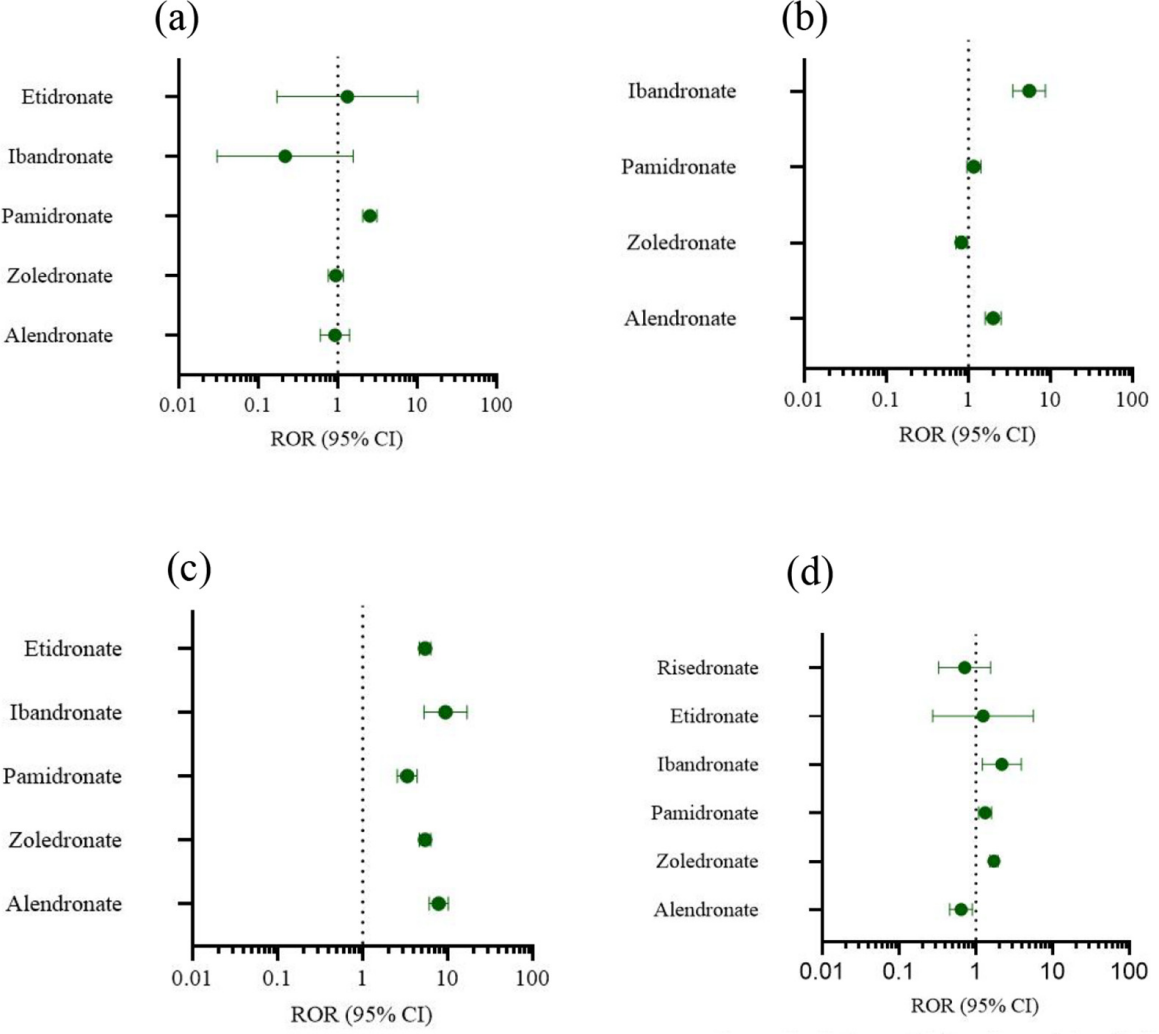
Forest plot of ROR. **(a)** Various examinations. **(b)** Injuries, poisoning and operational complications. **(c)** Musculoskeletal and connective tissue diseases. **(d)** Systematic diseases and reactions at the administration site.

Two data mining techniques, specifically the proportional reporting ratio (ROR) and the Bayesian confidence propagation information component neural network (IC), are employed for conducting disproportionate analysis. All other drugs/events were documented for comparative analysis. Statistical findings indicate that pamidronate exhibited significant signals across various examinations (Figure 3a). Alendronate and ibandronate demonstrated high signal intensity for incidents such as diverse injuries, poisoning, and surgical complications (Figure 3b). For various musculoskeletal and connective tissue diseases, all bisphosphonates express strong signals, especially alendronate (Figure 3c). Systemic diseases and various reactions at the administration site are strongly signaled by zoledronate, pamidronate and ibandronate (Figure 3d).

## 4 Discussion

The widespread use of bisphosphonate has been supported by numerous studies demonstrating its efficacy, yet also revealing an increasing number of adverse reactions. This study represents the largest and most comprehensive analysis to date of the effectiveness and safety profile of bisphosphonates in the pediatric population. Data gathered from existing research and the FAERS database indicate that bisphosphonate exhibits superior therapeutic benefits compared to the control drug, but also carries certain safety risks.

This study is grounded in clinical observations indicating that glucocorticoids are implicated in the induction of osteoporosis and are frequently associated with adverse effects related to osteonecrosis. Notably, some pediatric patients exhibit both conditions simultaneously (32). Although the efficacy of bisphosphonates in treating pediatric osteonecrosis has been examined, it is unfortunate that our literature search concerning glucocorticoid-induced bone diseases, incorporating terms related to bone infarct, did not produce relevant studies (33). As a result, this study is limited to exploring glucocorticoid-induced osteoporosis.

Despite children's robust bone reconstruction ability (34), there remains a concerning and increasing prevalence of vertebral fractures in this population. Research indicates that approximately 10% of children screened within the first year exhibit vertebral fractures, with nearly half of these cases being asymptomatic (35). Furthermore, children experiencing stunted growth, older children with limited residual growth potential, and those at ongoing risk for compromised bone health are less likely to undergo vertebral body remodeling following a spinal fracture. Failure to initiate treatment promptly may result in lasting deformities of the vertebral bodies (36). Bisphosphonates are commonly utilized in managing osteoporosis induced by hormone therapy in pediatric patients. Previous research has demonstrated the effectiveness of bisphosphonates in enhancing bone density and preventing osteolysis, aligning with the findings of our study (37–39).

In this meta-analysis, a synthesis of 14 studies was conducted to assess the effectiveness and safety of bisphosphonate therapy in managing steroid-induced osteoporosis in pediatric patients. Notably, this study represents a novel contribution by specifically focusing on children who have received hormone therapy, distinguishing it from prior analyses. Our analysis included six randomized controlled trials involving 572 patients. Given the unique physiological functions of children and the specific characteristics of drug-induced osteoporosis, our study may offer valuable insights for the management of osteoporosis resulting from hormone therapy in pediatric patients. In this research, a random effect model was utilized to compare the efficacy of bisphosphonate with that of the control group, revealing a significant improvement in bone mineral density (BMD) and BMD *Z*-scores with bisphosphonate treatment. Furthermore, a fixed effect model was employed for analysis, indicating no significant disparity in the efficacy of bisphosphonate compared to the control group post-fracture treatment. Additionally, concerning safety considerations, the incidence of adverse reactions was found to be higher in children treated with bisphosphonate compared to those in the control group.

According to Kan SL et al.'s study, bisphosphonate therapy is more effective in preventing and treating osteoporosis in rheumatic patients compared to calcium, vitamin D, or calcitonin (40). Consistent with prior research findings, our study also demonstrates that bisphosphonates are efficacious in preventing and treating hormone-induced alterations in bone mineral density. However, our analysis reveals no statistically significant disparity in fracture risk between the treatment and control groups (41). Prior research has primarily focused on adult patients, prompting our investigation into the applicability of these findings to children. Subsequent studies have indicated that bisphosphonates may offer greater benefits compared to active vitamin D3 analogues in mitigating the risk of glucocorticoid-induced fractures (42). Furthermore, a number of studies have demonstrated the impact of bisphosphonates in mitigating the likelihood of fractures (16, 43–45). However, our findings do not align with this assertion, as it is posited that certain bisphosphonates exhibit a diminished efficacy in reducing fracture risk compared to control group preparations (42), likely attributable to the combined effects of various bisphosphonates. Consequently, further research is warranted to explore the efficacy of bisphosphonates in fracture treatment.

The potential adverse effects of bisphosphonates have garnered significant attention. These effects encompass gastrointestinal disturbances, musculoskeletal discomfort, and acute phase reactions. Additionally, in rare instances, bisphosphonates may precipitate atrial fibrillation, atypical fractures, delayed fracture healing, osteonecrosis of the jaw, hypersensitivity reactions, and renal impairment (46). In the research conducted by Edwards BJ et al., a notable temporal association was observed between bisphosphonate use and atypical femoral fractures, with the incidence of fractures also demonstrating a significant correlation with the duration of bisphosphonate therapy. Furthermore, several studies have reported instances of induced fractures and osteosclerosis in pediatric populations (47–49). Our study observed a significant risk of adverse reactions associated with bisphosphonates in comparison to the control group when used for treating glucocorticoid-induced osteoporosis in Pediatric Patients. To enhance our understanding of the adverse reaction risk of bisphosphonates and address the absence of specific adverse reactions in the Meta analysis, we incorporated cases from the FAERS database. The majority of case reporters are professionals, thereby enhancing the credibility of the data. Our analysis revealed that a significant proportion of patients experienced adverse reactions within one month of initiating bisphosphonate therapy, thereby strengthening the association between adverse reactions and bisphosphonates to a certain degree. This also indicates a likelihood of adverse reactions occurring in the short term, underscoring the importance of vigilant monitoring for any abnormal patient responses. To mitigate potential adverse reactions, we conducted a comprehensive analysis of adverse events categorized by System Organ Class (SOC). Specifically, we identified four prominent categories of adverse reactions: various examinations, musculoskeletal and connective tissue diseases, injuries, poisoning and operational complications, and systemic diseases and reactions at the administration site. Notably, within the FAERS database, various examinations encompassed endocrine, cardiovascular, blood, and other examinations exhibiting abnormal values. Based on the statistical findings, it was determined that Pamidronate showed notable indications in the category of various examinations for adverse reactions. Previous literature has documented adverse reactions associated with bisphosphonates, including hypocalcemia and secondary hyperparathyroidism. Our study indicates that pamidronate is particularly pertinent to these atypical laboratory findings (50). Various injuries, poisonings, and operational complications primarily encompass fractures, medication errors, and other accidents, with alendronate and ibandronate showing high signal strength for such incidents. Musculoskeletal and connective tissue diseases are predominantly associated with arthritis, osteonecrosis, muscle weakness, and related conditions. All bisphosphonates, particularly alendronate, have exhibited robust signals, which we attribute to the constraints of the FAERS database, which cannot clearly establish the causal relationship between diseases and adverse reactions. The high prevalence of bisphosphonate use in diseases commonly treated with glucocorticoids underscores the significance of these findings. Alendronate constitutes 84% of oral bisphosphonates, as reported in the literature (51). Systemic diseases and various reactions at the administration site, such as injection site reactions, fever, treatment ineffectiveness, and other events, are prominently observed in the adverse event profiles of zoledronate, pamidronate, and ibandronate. The intravenous administration of bisphosphonates may result in a transient acute phase reaction characterized by symptoms such as bone and muscle pain, fever, myalgia, fatigue, and lymphopenia. The severity of these reactions may be dose-dependent and should be carefully monitored during the infusion of these medications (52).

While our study demonstrated the safety and efficacy of bisphosphonates for treating osteoporosis induced by hormone therapy in children, we acknowledge that potential biases may have arisen due to variations in factors such as route of administration, dosage, disease management, gender, and duration of treatment. Furthermore, our research primarily involves a comparison of bisphosphonates with a control group, thereby limiting our ability to make definitive conclusions regarding individual drug efficacy. A significant constraint of utilizing the FAERS database is the incomplete data and potential for selection and reporting biases (53), which hinders our ability to accurately determine the incidence of adverse reactions associated with each specific drug. Moreover, the causal relationship between bisphosphonates and adverse reactions may be uncertain, and there is a possibility of duplicate reports. Therefore, further high-quality research is necessary to thoroughly investigate the safety profile of bisphosphonates.

In summary, our research offers recommendations for managing osteoporosis fatalities resulting from hormone therapy in pediatric patients. While bisphosphonates demonstrate notable effectiveness, they also carry inherent risks of adverse reactions. Therefore, when choosing medications, a comprehensive evaluation should be conducted to mitigate potential risks by regulating the duration of drug administration.

## 5 Conclusion

This meta-analysis demonstrates that bisphosphonates can enhance bone mineral density (BMD) and BMD Z scores in Pediatric patients with GCs-induced osteoporosis. However, it also carries a risk of adverse reactions, highlighting the importance of considering this factor in clinical drug selection. Nevertheless, due to limitations in the current research, further evaluation through high-quality randomized controlled trials is warranted.

## Data Availability

Publicly available datasets were analyzed in this study. This data can be found here: These data can be found at: (1) https://research.cchmc.org/aers/explore.jsp (2) https://fis.fda.gov/sense/app/95239e26-e0be-42d9-a960-9a5f7f1c25ee/sheet/7a47a261-d58b-4203-a8aa-6d3021737452/state/analysis.
